# Determinants of Incidental Physical Activity During Pregnancy in Urban South India: Evidence of Volume Without Structure in a Community-Based Cross-Sectional Study

**DOI:** 10.7759/cureus.110843

**Published:** 2026-06-14

**Authors:** Aadithya D., Manoj P., Rajesh Kannan, Dorthy V., Sahanaa Yuvaraja

**Affiliations:** 1 Department of Community Medicine, Chettinad Hospital and Research Institute, Chettinad Academy of Research and Education, Kelambakkam, IND; 2 Department of Community Medicine, Sri Venkateshwaraa Medical College Hospital and Research Centre, Puducherry, IND

**Keywords:** antenatal counselling, determinants, physical activity, ppaq, pregnancy, urban india

## Abstract

Background

It is recommended that women engage in physical activity (PA) during pregnancy, as it is important for both maternal and fetal health; however, in urban Indian settings, this is mostly in the form of everyday domestic and transport-related tasks rather than planned exercise. The factors underlying this pattern (sufficient activity volume but a lack of structured exercise) are not well known.

Methodology

A cross-sectional study was conducted in the community among pregnant women (second/third trimester) from six urban field practice areas of Chettinad Hospital in Chengalpattu district, Tamil Nadu, India. Multistage sampling was used. The Pregnancy Physical Activity Questionnaire (PPAQ), which is validated and classifies participants as active (≥150 minutes/week, as recommended by the WHO), was used to measure PA. Activity domains were further categorized as structured (exercise/sports) and incidental (household/transport-related) activity. A validated Physical Activity Knowledge questionnaire consisting of 20 items was used to assess knowledge about PA. The chi-square test was used to analyze associations, and binary logistic regression was used to identify independent determinants.

Results

The average age of the subjects was 26.5 ± 3.8 years. The majority (*n* = 186, 92.5%) reported meeting the activity threshold recommended by the WHO, but all the activity reported was incidental, with no structured fitness practices. The mean total PA was 303.9 ± 102.1 minutes/week, most of which was from household and transport-related activities. Levels of knowledge were low (*n* = 96, 47.8%) and moderate (*n* = 101, 50.2%), with very low awareness of danger signs and contraindications to exercise. Multivariable analysis revealed that higher educational status (adjusted odds ratio (AOR) = 3.41; 95% confidence interval (CI) 1.24-9.38) and better knowledge (AOR = 4.12; 95% CI 1.18-14.37) were significant factors associated with being physically active. There were positive trends in the case of prior exercise, and no significant association was seen for counseling.

Conclusions

Most women had adequate levels of PA during pregnancy, but there was a lack of structured exercise, suggesting an issue with the quality of PA. Increasing awareness, promoting structured exercise practices, and strengthening antenatal counseling with specific instructions could help address this gap.

## Introduction

Women should engage in physical exercise during gestation to enhance the health of both the mother and the fetus. The American College of Obstetricians and Gynecologists (ACOG) and the World Health Organization (WHO) recommend that pregnant women who are not already engaged in moderate-intensity aerobic physical activity (PA) perform at least 150 minutes/week, unless contraindicated [1,2]. Pregnancy-related PA has been linked to better psychological health, a lower risk of gestational diabetes mellitus, excessive gestational weight gain, and hypertensive disorders [3,4]. However, there is still a lack of global adherence to these guidelines, especially in low- and middle-income countries (LMICs). Research from India indicates that only a small percentage of pregnant women reach the recommended levels of PA [5,6].

An important distinction exists between the quantity and quality of PA during pregnancy. In urban Indian settings, women often engage in substantial PA through household work and routine mobility. While such activities contribute to overall energy expenditure and are captured by validated tools such as the Pregnancy Physical Activity Questionnaire (PPAQ) [7], they differ from structured exercise in terms of activity domain and purpose. Indeed, women may appear to be achieving recommended activity levels based on duration but not on purposeful, moderate-intensity exercise [8,9]. This discrepancy highlights a difference between total activity volume and the composition of activity domains.

A variety of factors are involved in the determination of PA during pregnancy. Healthcare-related factors are also important, especially the type and quality of antenatal counseling, which is sometimes limited to general advice only in many settings, with no guidance on the type, intensity, and safety of exercise [10,11]. Sociocultural beliefs that encourage rest and restrict activity during pregnancy further contribute to reduced engagement in structured PA [12,13].

There is limited community-based evidence from urban South India examining PA patterns during pregnancy, particularly the relative contribution of incidental and structured activity domains. Understanding the composition of PA and the factors associated with achieving recommended activity levels is important for designing effective antenatal interventions. For the purpose of this study, structured exercise refers to purposeful sports- or exercise-domain activities captured by the PPAQ, whereas incidental activity refers to activity accumulated through household, caregiving, transportation, and occupational tasks. The term *volume without structure* describes a pattern in which recommended activity-duration thresholds are achieved primarily through incidental activity in the absence of reported structured exercise. The term *quality of physical activity* is used descriptively to denote the composition of activity domains rather than the physiological or clinical effectiveness of activity.

The primary objective of this study was to assess the composition of PA among pregnant women in an urban area of Chengalpattu district, with particular emphasis on the relative contribution of incidental and structured activity domains. The secondary objectives were to assess knowledge regarding PA during pregnancy and to identify factors associated with achieving WHO-recommended PA levels during pregnancy.

## Materials and methods

Study design and setting

A community-based cross-sectional study was conducted from June 2024 to December 2025 in six urban field practice areas (Sholinganallur, Semmancheri, Karapakkam, Injambakkam, Thoraipakkam, and Okkiyam) attached to Chettinad Hospital and Research Institute, Chengalpattu district, Tamil Nadu.

Study participants

Pregnant women aged ≥18 years in the second (≥14 weeks) or third trimester (<42 weeks), residing in the study area, and without contraindications to PA as per American College of Obstetricians and Gynecologists (ACOG) guidelines [2], were included. Women with pre-existing diabetes mellitus, hypertension, multiple gestation, or placenta previa were excluded.

Sample size and sampling technique

The sample size was calculated based on a prevalence of 43.7% moderate PA [14], using the formula *n* = 4*pq*/*d*², with a 95% confidence interval (CI) and 7% absolute precision, yielding a minimum sample size of 193, which was rounded to 200 to account for non-response. A total of 201 participants were enrolled. A multistage participant identification approach was used. In the first stage, six urban field practice areas attached to the institution were selected purposively. In the second stage, eligible pregnant women were identified through line listing using ASHA and ANM registers. In the final stage, eligible women were approached consecutively and enrolled until the required sample size was achieved.

Data collection

Data were collected through face-to-face interviews using a pre-tested semi-structured questionnaire. PA was assessed using the PPAQ [7], a validated tool measuring activity across household/caregiving, occupational, transportation, and sports/exercise domains. Activity was expressed in MET-hours/week. The PPAQ was administered through interviewer-based interviews. For each activity item, participants reported the average time spent in the activity. Activities were grouped according to the standard PPAQ domains (household/caregiving, occupational, transportation, and sports/exercise). For the WHO PA classification, the cumulative duration of moderate-intensity activities reported in the questionnaire was calculated in minutes per week. Participants achieving at least 150 minutes/week of moderate-intensity activity were classified as physically active, while those reporting less than 150 minutes/week were classified as inactive according to WHO recommendations [1]. Participants were classified as active (≥150 minutes/week of moderate-intensity activity, per WHO) or inactive (<150 minutes/week). Activity composition was further categorized as structured (presence of sports/exercise domain activity) or incidental (absence of sports/exercise activity, with activity derived from household, transport, and caregiving domains). Knowledge regarding PA during pregnancy was assessed using a validated 20-item questionnaire and categorized as poor (<50%), moderate (50%-75%), or good (>75%).

Statistical analysis

Microsoft Excel was used to enter the data, and IBM SPSS Statistics version 21.0 (IBM Corp., Armonk, NY) was used for analysis. Frequencies and percentages are used to display categorical variables, whereas mean ± standard deviation is used to display continuous variables. Fisher’s exact test or Pearson’s chi-square test was used, depending on the situation, to evaluate associations between participant characteristics and PA status (active vs inactive based on WHO-recommended activity levels). To identify factors independently associated with achieving WHO-recommended PA levels, binary logistic regression was performed. The variables used in the model were educational status, knowledge level, counseling, previous exercise, body mass index (BMI) category, and parity. As only 15 participants were classified as inactive, the number of outcome events was limited relative to the number of predictors included in the model. Therefore, the logistic regression analysis should be considered exploratory, and the adjusted odds ratios interpreted with caution.

Ethical considerations

The Chettinad Hospital and Research Institute Institutional Human Ethics Committee (IHEC) approved the study. Confidentiality was upheld, and all subjects provided written informed consent.

## Results

Two hundred and one participants were recruited for the study. They were pregnant women (mean age 26.5 ± 3.8 years). Most were graduates (*n* = 95, 47.3%) or postgraduates (*n* = 24, 11.9%), and the large majority were homemakers (*n* = 177, 88.1%). Nearly all were married (*n* = 200, 99.5%) and reported planned pregnancies (*n* = 194, 97.0%). Of 201 participants, 186 (92.5%) met the WHO ≥150 minutes/week volume threshold (classified as *active*), and 15 (7.5%) did not (*inactive*).

Educational attainment was the only sociodemographic variable significantly associated with PA status (χ² = 13.58, *P* = 0.012). Age, occupation, marital status, and spouse occupation showed no significant associations (all *P* > 0.05). Findings are presented in Table 1.

**Table 1:** Sociodemographic characteristics and association with physical activity status (N = 201). Active = ≥150 minutes/week of moderate-intensity activity per World Health Organization (WHO); inactive = <150 minutes/week. χ² = chi-square test. ^*^P < 0.05 - Statistically significant.

Variables	*n* (%)	Active (*n* = 186)	Inactive (*n* = 15)	*χ*²	*P*-value
Age (years), mean ± SD	26.5 ± 3.8	26.3 ± 3.5	27.9 ± 5.1	0.879	0.380
Age category: χ² (2)				1.21	0.541
18-24 years	61 (30.3)	55 (29.6)	6 (40.0)		
25-30 years	93 (46.3)	89 (47.8)	4 (26.7)		
>30 years	47 (23.4)	42 (22.6)	5 (33.3)		
Educational status: χ² (5)				13.58	0.012*
Illiterate	1 (0.5)	0 (0.0)	1 (6.7)		
Middle school	2 (1.0)	2 (1.1)	0 (0.0)		
High school	67 (33.3)	62 (33.3)	5 (33.3)		
Intermediate/Diploma	12 (6.0)	10 (5.4)	2 (13.3)		
Graduate	95 (47.3)	89 (47.8)	6 (40.0)		
Professional/Postgraduate	24 (11.9)	23 (12.4)	1 (6.7)		
Occupation: χ² (3)				3.71	0.821
Unemployed (Housewife)	177 (88.1)	162 (87.1)	15 (100.0)		
Skilled/Unskilled worker	4 (2.0)	2 (1.1)	0 (0.0)		
Clerical/Shop/Farm	3 (1.5)	3 (1.6)	0 (0.0)		
Semi-professional/Professional	17 (8.5)	17 (9.1)	0 (0.0)		
Marital status: χ² (1)				0.08	0.776
Married	200 (99.5)	185 (99.5)	15 (100.0)		
Living separately	1 (0.5)	1 (0.5)	0 (0.0)		
Spouse occupation: χ² (3)				2.14	0.837
Unemployed	1 (0.5)	1 (0.5)	0 (0.0)		
Unskilled/Semiskilled	124 (61.7)	119 (64.0)	5 (33.3)		
Clerical/Shop/Farm	23 (11.4)	21 (11.3)	2 (13.3)		
Semi-professional/Professional	53 (26.3)	45 (24.2)	8 (53.3)		

Total physical activity (TPA) ranged from 36 to 600 minutes/week (mean 303.9 ± 102.1 minutes/week; median 270 minutes/week). By WHO volume criteria, 186 (92.5%) were classified as active. However, a critical qualitative finding was the complete absence of structured exercise: no participant reported any activity in the sports/exercise domain of the PPAQ. All PA was incidental (*n* = 201, 100%), comprising household tasks, transportation-related walking, and caregiving. No participant engaged in vigorous-intensity exercise. Activity by intensity and domain is presented in Table 2.

**Table 2:** Physical activity by intensity and domain, and structured versus incidental classification (N = 201). Structured activity = any activity in the sports/exercise domain of the PPAQ; incidental activity = activity derived exclusively from household, transport, and caregiving domains. No participant reported structured exercise. PPAQ, Pregnancy Physical Activity Questionnaire

Activity category	Participants engaged, n (%)	Participants not engaged, n (%)	Mean (SD) (minutes/week)
Intensity			
Light intensity	200 (99.5)	1 (0.5)	158.8 ± 118.0
Moderate intensity	201 (100.0)	0 (0.0)	145.1 ± 85.6
Vigorous intensity	0 (0.0)	201 (100.0)	0.0
Domain			
Household/caregiving	194 (96.5)	7 (3.5)	-
Transportation	197 (98.0)	4 (2.0)	-
Recreational/leisure	192 (95.5)	9 (4.5)	-
Occupational	133 (66.2)	68 (33.8)	-
Structured vs. incidental			
Structured exercise (sports/exercise domain)	0 (0.0)	201 (100.0)	0.0
Incidental activity only (household + transport)	201 (100.0)	0 (0.0)	303.9 ± 102.1

Given the absence of structured activity across all participants, the WHO volume-based *active* classification reflects achievement of recommended activity volume but not participation in structured exercise. The significance of this distinction for public health communications and research is explored later.

The overall knowledge was poor among 96 (47.8%) participants, moderate among 101 (50.2%) participants, and good among 4 (2.0%) participants. Walking (196, 97.5%) and general maternal importance (*n* = 197, 98.0%) had the highest rates of awareness, while other acceptable activities, such as cycling (*n* = 39, 19.4%) and running (*n* = 24, 11.9%), had lower rates. Safety literacy was very limited: none of the participants were aware of all danger signs, and awareness of individual danger signs was low (vaginal bleeding: 11 (5.5%) and amniotic fluid leakage: 3 (1.5%)). All 201 (100%) participants were categorized as having poor knowledge of danger signs and contraindications. The detailed responses are presented in Table 3.

**Table 3:** Knowledge items regarding physical activity during pregnancy (N = 201). Danger sign awareness was critically deficient across all participants; all 201 participants were classified as having inadequate knowledge of contraindications and danger signs.

Knowledge item	Correct/Yes, *n* (%)	Incorrect/No, *n* (%)
Exercise types known as safe		
Walking	196 (97.5)	5 (2.5)
Breathing and pelvic floor exercises	164 (81.6)	37 (18.4)
Cycling	39 (19.4)	162 (80.6)
Running	24 (11.9)	177 (88.1)
Perceived maternal benefits		
General importance for the mother	197 (98.0)	4 (2.0)
Reduces Cesarean section rate	162 (80.6)	39 (19.4)
Shortens duration of labor	147 (73.1)	54 (26.9)
Reduces fatigue and stress	141 (70.1)	60 (29.9)
Reduces the risk of diabetes mellitus	124 (61.7)	77 (38.3)
Reduces risk of Preeclampsia	121 (60.2)	80 (39.8)
Perceived fetal benefits		
General importance for the fetus	189 (94.0)	12 (6.0)
Reduces risk of preterm birth	133 (66.2)	68 (33.8)
Reduces fetal distress during labor	131 (65.2)	70 (34.8)
Reduces risk of macrosomia/LBW	125 (62.2)	76 (37.8)
Safety knowledge (unsafe practices correctly identified)		
Exercising while dehydrated	191 (95.0)	10 (5.0)
Long-distance running	190 (94.5)	11 (5.5)
Marked straining exercises	188 (93.5)	13 (6.5)
Supine position exercise	158 (78.6)	43 (21.4)
Danger signs recognized (critically low)		
Calf pain	21 (10.4)	180 (89.6)
Vaginal bleeding	11 (5.5)	190 (94.5)
Amniotic fluid leaks	3 (1.5)	198 (98.5)

A statistically significant association was found between overall knowledge level and PA status (*P* < 0.01), with a greater proportion of active women having moderate or good knowledge compared with inactive women (102/186 vs. 2/15). Domain-specific knowledge scores (exercise types, maternal benefits, and fetal benefits) were not significantly associated with activity status (all *P* > 0.05). Knowledge of contraindications and danger signs could not be analyzed due to zero variance. Findings are presented in Table 4.

**Table 4:** Knowledge level and domain scores: association with physical activity status (N = 201). N/A, not applicable (all participants classified as inadequate; zero variance). χ² = chi-square test. *P < 0.05 - Statistically significant.

Variables	*n* (%)	Active (*n* = 186)	Inactive (*n* = 15)	*χ*²/*t*	*P*-value
Overall knowledge level: χ² (2)				-	<0.01*
Poor (<50%)	96 (47.8)	84 (45.2)	12 (80.0)		
Moderate (50%-75%)	101 (50.2)	99 (53.2)	2 (13.3)		
Good (>75%)	4 (2.0)	3 (1.6)	0 (0.0)		
Knowledge of exercise types: χ² (1)				1.57	0.211
Inadequate	151 (75.1)	142 (76.3)	9 (60.0)		
Adequate	50 (24.9)	44 (23.7)	6 (40.0)		
Knowledge of maternal benefits: χ² (1)				1.62	0.203
Inadequate	112 (55.7)	106 (57.0)	6 (40.0)		
Adequate	89 (44.3)	80 (43.0)	9 (60.0)		
Knowledge of fetal benefits: χ² (1)				0.00	0.974
Inadequate	108 (53.7)	100 (53.8)	8 (53.3)		
Adequate	93 (46.3)	86 (46.2)	7 (46.7)		
Knowledge of contraindications				-	N/A
Inadequate (all participants)	201 (100.0)	186 (100.0)	15 (100.0)		
Knowledge of danger signs				-	N/A
Inadequate (all participants)	201 (100.0)	186 (100.0)	15 (100.0)		

Family and friends were the primary source of information (*n* = 93, 46.3%), followed by health professionals (*n* = 89, 44.3%). Of the 201 participants, 113 (56.2%) received counseling on PA; however, the advice was generally limited to statements such as “walking is good” rather than being specific regarding intensity, duration, frequency, or safety precautions as recommended by healthcare professionals. This represents a counseling quality issue rather than a coverage issue. Access to a supportive environment was reasonably favorable (open space: *n* = 147, 73.1%; facility support: *n* = 103, 51.2%), and neither factor was significantly associated with PA status. Prior exercise before pregnancy was reported by 52 (25.9%) participants and was significantly associated with being physically active during pregnancy (χ² = 4.21, *P* = 0.040). These associations are presented in Table 5.

**Table 5:** Information sources, counseling, environmental access, and prior exercise: association with physical activity status (N = 201). Receipt of counseling was not significantly associated with active status (*P* = 0.568), consistent with inadequate counseling quality rather than insufficient coverage. χ² = Chi-square test. *P < 0.05 - Statistically significant.

Variables	*n* (%)	Active (*n* = 186)	Inactive (*n* = 15)	χ²/*t*	P-value
Primary source of information on physical activity					
Primary information source: χ² (3)				4.62	0.200
Family/Friends	93 (46.3)	84 (45.2)	9 (60.0)		
Health professionals	89 (44.3)	84 (45.2)	5 (33.3)		
Mass media	16 (8.0)	15 (8.1)	1 (6.7)		
Books/Other	3 (1.5)	3 (1.6)	0 (0.0)		
Receipt and quality of professional advice on physical activity					
Received health professional advice: χ² (1)				0.33	0.568
Yes	113 (56.2)	105 (56.5)	8 (53.3)		
No	88 (43.8)	81 (43.5)	7 (46.7)		
Environmental access					
Access to open spaces/sports fields: χ² (1)				1.11	0.291
Yes	147 (73.1)	138 (74.2)	9 (60.0)		
No	54 (26.9)	48 (25.8)	6 (40.0)		
Exercise facility support available: χ² (1)				0.82	0.365
Yes	103 (51.2)	96 (51.6)	7 (46.7)		
No	98 (48.8)	90 (48.4)	8 (53.3)		
Prior physical activity behavior					
Regular exercise before pregnancy: χ² (1)				4.21	0.040*
Yes	52 (25.9)	51 (27.4)	1 (6.7)		
No	149 (74.1)	135 (72.6)	14 (93.3)		

Achievement of WHO-recommended PA levels (active vs inactive) was used as the dependent variable in binary logistic regression after controlling for educational attainment, overall knowledge level, previous PA, counseling received, BMI category, and parity. The model was exploratory given the small inactive group (*n* = 15, 7.5%), and the results should be interpreted accordingly.

On multivariable analysis, higher educational attainment (graduate/postgraduate vs ≤high school: adjusted odds ratio (AOR) 3.41, 95% CI 1.24-9.38, *P* = 0.017) and a better overall knowledge level (moderate/good vs poor: AOR 4.12, 95% CI 1.18-14.37, *P* = 0.026) were independently associated with active status. Prior exercise before pregnancy showed a strong but non-significant trend (AOR 6.83, 95% CI 0.84-55.3, *P* = 0.072), likely limited by the small inactive group. Receipt of counseling (AOR 1.29, *P* = 0.681), BMI category (AOR 0.71, *P* = 0.561), and parity (AOR 0.88, *P* = 0.840) were not independently associated. Results are presented in Table 6.

**Table 6:** Multivariable logistic regression: independent determinants of active physical activity status (N = 201). Reference categories: education ≤high school; knowledge poor; counseling not received; prior exercise no; BMI normal; parity multiparous. Multivariable logistic regression test was used. *P < 0.05 - Statistically significant. AOR, adjusted odds ratio; BMI, body mass index

Variable	Category	AOR (95% CI)	*P*-value
Educational status	Graduate/PG vs. ≤High school	3.41 (1.24-9.38)	0.017
Overall knowledge	Moderate/Good vs. Poor	4.12 (1.18-14.37)	0.026
Prior exercise	Yes vs. No	6.83 (0.84-55.3)	0.072
Counseling received	Yes vs. No	1.29 (0.38-4.36)	0.681
BMI	Overweight/Obese vs. Normal	0.71 (0.22-2.28)	0.561
Parity	Primiparous vs. Multiparous	0.88 (0.27-2.89)	0.840

## Discussion

Although the study identified a complete absence of reported structured exercise, the determinant analysis was based on achievement of WHO-recommended activity volume and therefore reflects factors associated with overall activity levels rather than participation in structured exercise.

This study identifies an important discrepancy between the volume and the quality of PA during pregnancy. While the majority (n = 186, 92.5%) of participants achieved the recommended level of ≥150 minutes/week [1], none engaged in structured exercise, and all reported activity was incidental. This *volume without a structure* pattern suggests that classification by volume alone may not fully capture the composition of PA during pregnancy. This is consistent with other LMICs, where the majority of energy is spent on domestic and transport activities, with little involvement in structured exercise [8,9,15].

Higher knowledge levels were associated with active PA status in the present study. A major finding was the significant lack of safety knowledge; this was consistent with behavior change models that have shown that knowledge can lead to behavior [16,17]. This is poor awareness of contraindications and danger signs and suggests that there is a major gap in the antenatal education [18]. The absence of reported structured exercise may be influenced by fear, uncertainty, or limited awareness regarding exercise during pregnancy.

Educational attainment was the only sociodemographic factor significantly associated with active PA status in the present study aside from the influence of knowledge [6,10], indicating that education may affect health behavior beyond knowledge, perhaps through increased health literacy and decision-making autonomy. Prior exercise before pregnancy also showed a positive association, indicating the role of behavioral continuity [19]. Given the cross-sectional nature of the study and the limited number of inactive participants, these associations should be interpreted as exploratory and should not be considered evidence of causality.

Despite more than half of the participants reporting receipt of counseling, no significant association was observed between counseling and PA status. This finding may suggest that the counseling received by participants was predominantly general in nature and may not have included detailed guidance regarding exercise type, intensity, duration, or safety; thus, it may indicate opportunities to strengthen the specificity of antenatal PA counseling. However, counseling quality was not formally assessed in the present study [12,20]. Additionally, the complete absence of structured exercise despite reasonable environmental access suggests that sociocultural factors and family norms may contribute to PA behaviors; however, these factors were not directly measured in the present study and warrant further investigation [11].

This study provides community-based evidence on both the volume and composition of PA using a validated domain-specific tool [7], allowing differentiation between incidental and structured activity. However, several limitations should be considered when interpreting these findings. The cross-sectional design precludes determination of temporal or causal relationships between the identified factors and PA status. PA and knowledge were assessed using self-reported measures and may therefore be subject to recall bias and social desirability bias. The study did not assess maternal or fetal health outcomes and therefore cannot determine the clinical effects of incidental versus structured PA. Similarly, although no participant reported structured exercise, the present findings do not permit conclusions regarding the comparative effectiveness of different activity domains. The six field practice areas were selected purposively, and eligible participants were enrolled consecutively, which may have introduced selection bias and may limit generalizability beyond similar urban settings. In addition, classification of activity status involved the application of WHO activity recommendations to PPAQ-derived activity data, which may have resulted in some degree of misclassification. Finally, only a small proportion of participants were classified as inactive, limiting statistical power and rendering the multivariable regression analysis exploratory in nature.

Overall, the findings indicate that antenatal PA promotion may benefit from more specific guidance regarding exercise type, frequency, duration, and safety during pregnancy, with emphasis on safety literacy and differentiation between incidental and structured activity. Increased uptake of family-inclusive counseling and preconception interventions can further enhance uptake. Further studies are needed to better understand the determinants of structured exercise during pregnancy using objective activity measures and larger sample sizes.

## Conclusions

This study reveals a distinct *volume-quality paradox* in antenatal PA: the majority of women exceeded the WHO-recommended amount of PA, but none reported participation in structured exercise activities. Educational attainment and overall knowledge were important factors significantly associated with active PA status, while receipt of counseling was not independently associated with achieving recommended PA levels. Further research is needed to better understand the content and effectiveness of antenatal counseling related to PA. A lack of safety literacy was universal, reinforcing the need for safety education in maternal health.

These results suggest that volume-based measures alone may not fully characterize the composition of physical activity during pregnancy. To better support antenatal physical activity promotion, greater emphasis may be placed on providing specific exercise guidance, safety-focused counseling, and early behavioral interventions, while further research is needed to understand the implications of different activity patterns during pregnancy and identify effective strategies for promoting appropriate physical activity behaviors.
